# High throughput nano-liter RT-qPCR to classify soil contamination using a soil arthropod

**DOI:** 10.1186/1471-2199-12-11

**Published:** 2011-03-01

**Authors:** Muriel E de Boer, Sandra Berg, Martijn JTN Timmermans, Johan T den Dunnen, Nico M van Straalen, Jacintha Ellers, Dick Roelofs

**Affiliations:** 1 VU University Amsterdam, Department of Ecological Science, De Boelelaan 1085, 1081 HV, Amsterdam, The Netherlands; 2 Imperial College London, Faculty of Natural Sciences, Department of Ecology & Evolution, Exhibition Road, London SW7 2AZ, UK; 3 LUMC, Leiden Genome Technology Center, Einthovenweg 20, 2333 ZC Leiden, The Netherlands

## Abstract

**Background:**

To incorporate genomics data into environmental assessments a mechanistic perspective of interactions between chemicals and induced biological processes needs to be developed. Since chemical compounds with structural similarity often induce comparable biological responses in exposed animals, gene expression signatures can serve as a starting point for the assessment of chemicals and their toxicity, but only when relevant and stable gene panels are available. To design such a panel, we isolated differentially expressed gene fragments from the soil arthropod *Folsomia candida*, a species often used for ecotoxicological testing. Animals were exposed to two chemically distinct compounds, being a metal (cadmium) and a polycyclic aromatic hydrocarbon (phenanthrene). We investigated the affected molecular responses resulting from either treatment and developed and validated 44 qPCR assays for their responses using a high throughput nano-liter RT-qPCR platform for the analysis of the samples.

**Results:**

Suppressive subtractive hybridization (SSH) was used to retrieve stress-related gene fragments. SSH libraries revealed pathways involved in mitochondrial dysfunction and protein degradation for cadmium and biotransformation for phenanthrene to be overrepresented. Amongst a small cluster of SSH-derived cadmium responsive markers were an inflammatory response protein and an endo-glucanase. Conversely, cytochrome P450 family 6 or 9 was specifically induced by phenanthrene. Differential expressions of these candidate biomarkers were also highly significant in the independently generated test sample set. Toxicity levels in different training samples were not reflected by any of the markers' intensity of expressions. Though, a model based on partial least squares differential analysis (PLS-DA) (with RMSEPs between 9 and 22% and R^2^s between 0.82 and 0.97) using gene expressions of 25 important qPCR assays correctly predicted the nature of exposures of test samples.

**Conclusions:**

For the application of molecular bio-indication in environmental assessments, multivariate analyses obviously have an added value over univariate methods. Our results suggest that compound discrimination can be achieved by PLS-DA, based on a hard classification of the within-class rankings of samples from a test set. This study clearly shows that the use of high throughput RT-qPCR could be a valuable tool in ecotoxicology combining high throughput with analytical sensitivity.

## Background

In the field of environmental sciences a high throughput molecular research often called 'ecogenomics' [1,2] has evolved over the past 10 years. The current challenge for ecotoxicology is to benefit most from the outburst of molecular knowledge [3] initially mainly generated by microarray studies, later followed by expressed sequence tag (EST) sequencing and mapping (see for an overview [4]) which in turn is currently being followed up by next generation sequencing of cDNAs (RNA-Seq). Ideally, the integration of "omics" data with traditional ecotoxicological parameters will elucidate mechanistic networks that can be used to incorporate biomarkers in predictive quantitative models of adverse outcome pathways [5,6].

Traditionally, the ecotoxicological approach is focused on the effects of different concentration levels of chemical compounds on organisms, rather than on the molecular and cellular mechanisms underlying these effects. In contrast, ecogenomics aims at studying genome-wide molecular biological processes in relation to toxicity and hence has a more mechanistic approach. Such an approach at the level of affected cellular processes and genetic response pathways may give new insights into the main hazards to human and environmental health, and may support the classification by hazard and the authorization of new and existing chemicals. In turn, this may aid the design of new highly selective environmental chemicals less hazardous for non-target species.

It is suggested that molecular biomarkers based on gene induction, in combination with conventional endpoints, can provide robust insight of the dose responses and mechanistic underlying effects of unknown chemical compounds [6]. Studies from research fields where this is a central premise, such as medicinal chemistry, have shown that structurally similar molecules have similar biological activities [7]. This so-called 'neighborhood behavior' [8] is validated by long experience and has led to rules-of-thumb such as "beta-lactams frequently possess antibacterial activity". However, also compelling examples of the lack of parallel between structural and biological similarity are known [7]. Using a genomics technology of high throughput quantitative PCR arrays, Vass et al. [9] tested 625 cytotoxic compounds for neighborhood behavior in human hepatic cells. The nature of the compounds ranged from pesticides to hormone mimickers to potential anti-cancer drugs, with a common characteristic molecular structure or 'scaffold' for each family. Eight out of twelve different molecular families showed correlation between scaffold and gene expression profile over the selected toxicity gene panel. Importantly, the authors conclude that the best markers for finding correlations between a library of molecular scaffolds and their general biological fingerprint would most probably not be those measuring toxicity. In other words, when testing compounds of such different nature, an initial 'molecular clustering' into predefined classes is required. For this task one needs to use a set of tailor-made markers. Only when such markers have been identified can we investigate toxicity levels and neighborhood behavior for a particular level of toxic effects.

In this study we explore the high throughput nano-liter RT-qPCR system of Fluidigm/Biomark as a novel next generation genomics tool to address whether compound-specific responses or even single biomarkers can be identified for two common environmental pollutants, the metal cadmium and the polycyclic aromatic hydrocarbon (PAH) phenanthrene, using the ecotoxicological model organism *Folsomia candida *(Collembola: Isotomidae). RT-qPCR has a higher dynamic range than microarrays, which also suffer from inaccurate mRNA quantification due to the fact that detection relies on probe hybridization. The Fluidigm/Biomark Dynamic Array is an innovative platform exceeding the possibilities of sample throughput of microarrays, with much more accurate mRNA quantification (see [10] for full details).

The studied chemicals were chosen as they have very different modes of action. Cadmium is known to induce a great variety of cellular effects, including its interaction with enzymes (e.g. [11]) and the induction of oxidative stress [12], while phenanthrene, a PAH formed by incomplete combustion of fossil fuels, is toxic because its lipid-soluble nature facilitates the traverse of cell membranes and similar barriers in the body (e.g. [11]) and can disrupt the integrity and functioning of these compartments. Kültz [13] describes potential stress sensing mechanisms that are based on lipid membrane damage and rearrangements, like for instance the activation of phospholipase A2, which leads to liberation of arachidonic acid from membranes. As a possible stress sensor, this may eventually induce a specific set of genes, related to xenobiotic biotransformation (e.g. [14]).

To acquire an initial collection of candidate markers, the 'open' technique of suppressive subtractive hybridization (SSH) [15] was used. Today the state of the art method for discovery and identification of specific stress-induced sequences is RNA-seq [16]. Less suited for picking up low abundant transcripts, but for the majority of cases RNA-seq by now surpassed the use of SSH. We then used high throughput RT-qPCR for transcriptional signature profiling of specific targets selected from the SSH libraries. A microarray platform was developed concurrently [17]. Still, the high accuracy of measurement and large dynamic range needed for analysis of differential expression patterns is yet only achieved by RT-qPCR (e. g. [16,18]). By using microfluidic high throughput qPCR chips [10,19], the quantitative assessment could be combined with a fairly large number of simultaneously run gene assays and samples. The Fluidigm platform [10] also has the advantage of a totally flexible chip setup which made it altogether a cost effective approach for applied transcriptional profiling. (See the methods section of this article for a brief overview of the system.)

We performed initial testing and validation of 44 markers in *F. candida *exposed to different concentrations of cadmium and phenanthrene in soil. We verified the analytical performance and applicability of the high throughput platform for eco-toxicogenomic application. Moreover, flexibility in test setup and low amounts of required input cDNA made this application very convenient for the analysis of our experiments.

## Results

### General library statistics

**Figure 1:**
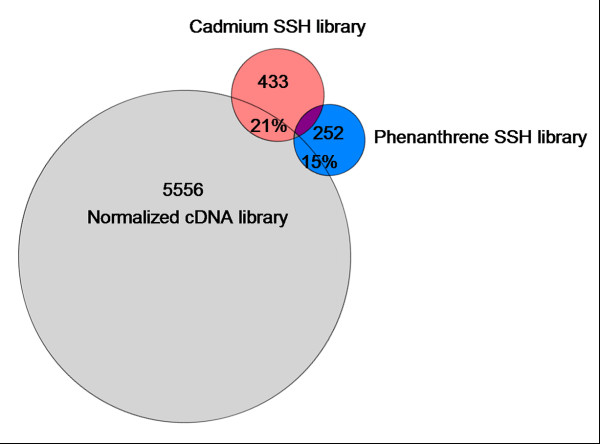
**Proportional Venn-diagram of library sizes of *Folsomia candida *exposed to cadmium and phenanthrene according to the number of assembled clusters**. Percentage cluster overlap between the suppressive subtractive hybridization (SSH) libraries for cadmium (21%) and phenanthrene (15%) with the normalized library Cluster totals were 433 for cadmium (red), 252 for phenanthrene (blue) and 5556 for the normalized library (see [20] for details).

A total of 960 clones were sequenced of both the cadmium and phenanthrene SSH library. The processing of the sequences is described in detail in Timmermans et al. [20]. Sequences can be accessed via GenBank [21] (Accession numbers: EV473060 - EV481745) or Collembase [22]. Summarizing, ESTs ranged between 98 and 691 base pairs (bp). SSH ESTs were assembled simultaneously with the cDNAs from a normalized cDNA library that was developed concurrently (see [20]) into unique gene objects (clusters) up to a length of 1432 bp. The cadmium library counted a total of 433 clusters, the phenanthrene library 252. The area-proportional Venn diagram (Figure 1; [23]) shows the relative size of each library and illustrates the number of clusters that contained clones from more than one library by the size-proportional overlapping areas: 21% of the total cadmium clusters (91) and 15% of the total phenanthrene clusters (39) overlapped with the normalized library. Furthermore, 26 clusters were identical between cadmium and phenanthrene, and 7 clusters contained clones from all three libraries [20]. Annotation levels of the libraries were 65% for cadmium and 52% for phenanthrene (Blastx analysis; non-redundant (nr) database; expect-value < 10^-5^); these levels are commonly observed for SSH libraries (e.g 56% in [24]; 55% in [25] and 30% in [26]).

### Gene Ontology enrichment analysis

#### CateGOrizer GO slims classification count

**Figure 2:**
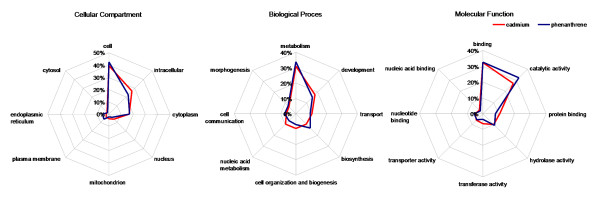
**Percentage of occurrence of generic GO Slim ancestor terms in SSH libraries of *Folsomia candida *exposed to cadmium and phenanthrene**. Occurrences of GO-terms were counted by single counting of all GO-terms assigned to clusters in the cadmium (red) and phenanthrene (blue) suppressive subtractive hybridization (SSH) library, counted by CateGOrizer [27], Each of the eight corners of the radar plots represent an ancestor term (plots designed with Chart Tool [69]).

GO terms could be assigned to approximately 35% of all clusters using the Annot8r_blast2GO script (see [20] for details). GO term occurrence in the different libraries was calculated using the GO term classification counter CateGOrizer [27]. A summary of the results for both libraries is given in Figure 2, sorted by root class cellular component (CC), biological process (BP) and molecular function (MF). Within each root class differences were seen especially in CC. The term intracellular (GO:0005622) was more frequent in cadmium while plasma membrane (GO:0005886) and endoplasmic reticulum (GO:0005783) ocurred more frequently in phenanthrene. In BP, development (GO:0007275), cell organization and biogenesis (GO:0016043), and nucleic acid metabolism (GO:0006139) were more frequent in cadmium, while metabolism, (GO:0008152) and biosynthesis (GO:0009058) were more frequent in phenanthrene. In MF, protein binding (GO:0005515) and transferase activity (GO:0016740) were more abundant in the cadmium library, while catalytic activity (GO:0003824) was more abundant in the phenanthrene library.

#### GOEAST Enrichment analysis

The enrichment status of the SSH libraries was further interpreted using another online tool; GOEAST for customized analysis [28]. All GO terms available for *F. candida *using both SSH libraries and the normalized library, were used as basic background for the classification mapping. Using the Multi GOEAST tool to compose directed a-cyclic graphs (DAG) hierarchical maps for each root class, the results for the two stresses could be compared easily in a visually attractive way (Additional file 1, 2, 3 for CC, BP and MF respectively).

To compare the relative abundance between the two SSH libraries, significantly enriched (p < 0.01) GO terms from the GOEAST analysis, were again imported in CateGOrizer for a counting within each collection; the phenanthrene, cadmium and the normalized unstressed set. Table 1 gives a summary of the terms with the highest log_2 _odds ratios (>2; GOEAST analysis), which is a measure for the relative abundance of that particular term in the given dataset compared to a random situation. Also included are any terms that were interesting because they showed high difference in occurrence ratio between the phenanthrene and cadmium libraries (> 2; CateGOrizer analysis). This explorative approach shows the trends (+, -, 0; higher, lower and equally represented relative to the normalized unstressed library) of gene classes that are changed most in at least one of the SSH libraries, which is actually a simplified representation of the DAG maps.

**Table 1:** Summary of GO term classes enriched in SSH libraries of cadmium and phenanthrene responsive genes

	**Term**	Cadmium*	Phenanthrene*	**GO IDs**				
Cellular Compartment	vacuolar membrane	**+**	**-**	GO:0005774	GO:0044437			
	mitochondrial inner membrane	**+**	**-**	GO:0005743	GO:0019866			
	mitochondrial respiratory chain	**+**	**-**	GO:0005746	GO:0070469			
	cis-Golgi network	**+**	**-**	GO:0005801				
	proteasome core complex	**+**	**-**	GO:0005839				
	integral to membrane of membrane fraction	**+**	**-**	GO:0000299				
	apical plasma membrane	**-**	**+**	GO:0016324	GO:0045177			
	protein-DNA complex	**-**	**+**	GO:0032993				
	cell-cell junction	**o**	**+**	GO:0005911				
	lipid particle	**o**	**+**	GO:0005811				
	microsome	**o**	**+**	GO:0005792				
	vesicular fraction	**o**	**+**	GO:0042598				
Biological Process	antibiotic metabolism and biosynthesis	**+**	**+**	GO:0016999	GO:0017000			
	drug metabolic process	**+**	**+**	GO:0017144				
	metabolic processess (phenol, amino acids)	**+**	**+**	GO:0006584	GO:0018958	GO:0006576	GO:0006575	
	response to alkaloid	**+**	**+**	GO:0043279				
	(di) terpenoid metabolic process	**+**	**o**	GO:0006721	GO:0016101			
	bone development, ossification	**+**	**o**	GO:0060348	GO:0001503			
	vitamin A metabolic process	**+**	**o**	GO:0006776	GO:0001523			
	drug transport	**+**	**o**	GO:0015893				
	tissue remodeling	**+**	**-**	GO:0048771				
	mitochondrion distribution, inheritance, organization	**+**	**-**	GO:0048311	GO:0000001	GO:0048308	GO:0007005	
	autophagy	**+**	**-**	GO:0006914				
	glyoxylate cycle	**+**	**-**	GO:0006097	GO:0046487			
	metabolism of alditol, polyol, glycerol, aldehyde, process	**+**	**-**	GO:0019751	GO:0006071	GO:0019400	GO:0006081	
	fat-soluble vitamin metabolic process	**+**	**-**	GO:0006775				
	cellular aerobic respiration	**+**	**-**	GO:0009060	GO:0045333			
	peripheral nervous system development	**+**	**-**	GO:0007422	GO:0007411			
	(canalicular) bile acid and bile salt transport	**o**	**+**	GO:0015721	GO:0015722			
	histidine meta- and catabolism	**o**	**+**	GO:0006548	GO:0009075	GO:0006547	GO:0009077	
	monocarboxylic acid transport	**o**	**+**	GO:0015718				
	response to purine, caffeine	**-**	**+**	GO:0014074	GO:0031000			
	iron ion transport	**-**	**+**	GO:0006826				
	olfactory chemosensory behavior	**-**	**+**	GO:0042048	GO:0007635			
	acute inflammatory response	**-**	**+**	GO:0002526				
	tRNA aminoacylation for protein translation	**-**	**+**	GO:0006418	GO:0043039			
	cellular amino acid catabolic process, activation	**-**	**+**	GO:0009063	GO:0043038	GO:0044270	GO:0009310	
	autophagic cell death	**-**	**+**	GO:0048102				
Molecular Function	N-(5-amino-5-carboxypentanoyl)-L-cysteinyl-D-valine synthase activity	**+**	**+**	GO:0050564				
	amino-acid racemase activity	**+**	**+**	GO:0047661				
	transition metal ion transmembrane transporter activity	**+**	**+**	GO:0046915				
	ATPase activity	**+**	**+**	GO:0043492	GO:0042626	GO:0015662	GO:0008553	
	L-ascorbic acid binding	**+**	**+**	GO:0031418				
	phosphopantetheine binding	**+**	**+**	GO:0031177				
	racemase and epimerase activity	**+**	**+**	GO:0016854	GO:0016855			
	hydrolase activity, acting on acid anhydrides, catalyzing transmembrane movement of substances	**+**	**+**	GO:0016820				
	oxidoreductase activity	**+**	**+**	GO:0016715				
	P-P-bond-hydrolysis-driven transmembrane transporter activity	**+**	**+**	GO:0015405				
	primary active transmembrane transporter activity	**+**	**+**	GO:0015399	GO:0015082	GO:0008514		
	copper ion binding	**+**	**+**	GO:0005507				
	histone acetyltransferase activity	**+**	**+**	GO:0004402				
	acyl carrier activity	**+**	**+**	GO:0000036				
	binding: amine, chloride ion	**+**	**o**	GO:0043176	GO:0043168	GO:0031404		
	ATPase activity	**+**	**o**	GO:0042625				
	ligand-gated (ion) channel activity	**+**	**o**	GO:0022834	GO:0015276			
	extracellular ligand-gated ion channel activity	**+**	**o**	GO:0005230				
	retinol dehydrogenase activity	**+**	**o**	GO:0004745				
	phosphoric diester hydrolase activity	**+**	**-**	GO:0008081				
	threonine-type endopeptidase activity	**+**	**-**	GO:0004298	GO:0070003			
	cation transmembrane transporter activity	**+**	**-**	GO:0008324	GO:0015075			
	binding: tetrapyrrole heme, vitamin, oxygen	**o**	**+**	GO:0020037	GO:0019842	GO:0019825	GO:0046906	
	oxidoreductase activity	**o**	**+**	GO:0016712	GO:0016705			
	iron ion binding	**o**	**+**	GO:0005506				
	monooxygenase activity	**o**	**+**	GO:0004497	GO:0004500			
	oxidoreductase activity	**-**	**+**	GO:0016709				
	endopeptidase (-inhibitor) activity	**-**	**+**	GO:0004866	GO:0004857	GO:0004177	GO:0004867	GO:0030414
	nucleoside binding	**-**	**+**	GO:0001882				
	aminoacyl-tRNA ligase activity	**-**	**+**	GO:0004812				
Terms with the highest log_2 _odds ratios (>2; GOEAST analysis), or large difference in occurrence ratio between the two SSH libraries (> 2; CateGOrizer analysis) are included in the table. * Trend of expression; higher (+), lower (-) or equal (o), based on term countings (Hu et al 2008) in the cadmium, phenanthrene and normalized library.								

Table 1 shows that within the category of cellular component and biological process there is only minor overlap in enrichment between the cadmium and phenanthrene libraries. For molecular function however, nearly half of the included terms are enriched in both libraries.

Genes related to membranes were found to be enriched in the category 'cellular component' in both the cadmium and phenanthrene sets. However, in the cadmium set this term points towards vacuolar membranes, the cis Golgi and the mitochondrial inner membrane, while in phenanthrene it most likely concerns the (apical) plasma membrane and the endomembrane structures of the endoplasmic reticulum (ER) (log_2 _odds ratio 1.7, Additional file 1). The smooth ER is the cellular structure in which cytochrome P450 and other biotransformation enzymes are located [14]. The GO terms microsomal and vesicular fraction, enriched in the phenanthrene library are formed from the smooth ER when the cell is homogenized. The GO term integral to membrane and membrane fraction, enriched in the cadmium library, also relate to fractions formed when cells are homogenized and do not necessarily refer to structures in the intact cells [29,30].

In the cadmium library, gene fragments coding for subunits of the proteasome were found. Proteasomes recognize, unfold, and digest protein substrates that have been marked for degradation [31].

Biological Processes enriched in both stress libraries are related to the biosynthesis of β-lactam antibiotics (penicillins and cephalosporins) [32]. Other enriched metabolic processes are those involved in biotransformation (phenanthrene), active transport and oxidative phosphorylation (cadmium). During the time course of this experiment a microarray platform was developed on the basis of the normalized and SSH gene libraries. The results found here were largely confirmed concurrently by analyses of microarray hybridizations [17,33]. Among the enriched molecular functions shared between libraries, we found enzymes related to the β-lactam pathway (e.g. N-(5-amino-5-carboxypentanoyl)-L-cysteinyl-D-valine synthase), active and facilitated transmembrane transport (including metals, organic anions and protons by ATPases in the respiratory chain), the binding of vitamin C and copper ions, and redox-related enzymes (hydrolases, dehydrogenases, oxidoreductases). Specific molecular functions for the cadmium library were for instance extracellular ligand-gated ion channel activity, retinol dehydrogenase activity, and cation transmembrane transporter activity. Specific for the phenanthrene library were iron binding, monooxygenase activity, and functions related to the translational process, nucleoside binding and aminoacyl-tRNA ligase activity.

### Quantitative PCR

Quantitative PCR markers were developed (Table 2, Additional file 4) for differentially expressed genes (see the materials section of this article for the criteria) that were selected based on hybridization differences between SSH probes hybridized on Southern dot blots of clones from the forward subtracted pools of each SSH. Melting curve analysis, PCR efficiency estimation as well as testing correlations between different regular qPCR and high throughput (Pearsons correlation coefficient > 0.97, 0.9 < slope <1.1) resulted in 44 technically validated and functional assays. Biological validation of the assays was performed using two different concentrations per compound. No significant differences were found between these concentrations and it was therefore decided to treat the exposed samples as one group per compound. The controls of both treatments, consisting of acetone solvent controls for phenanthrene and water controls for cadmium, were also considered as one group.

**Table 2:** Overview of differential qPCR assays and their importance and significances in the statistical analyses

**#**	**SSH origin**	**Collembase ID¹**	**Gene**	**PLS²**	**cadmium³**	**phenanthrene³**	**interaction³**
1	cadmium	Fcc00008	unknown function	VIP	***	ns	**
2	cadmium	Fcc00101	BCS1	VIP	***	*	ns
3	cadmium	Fcc00344	retinol dehydrogenase 11	VIP	***	*	ns
4	cadmium	Fcc00583	inflammatory response protein 6; viperin;	VIP	***	ns	***
5	cadmium	Fcc01017	Endo-1,3;1,4-beta-D-glucanase precursor	VIP	***	***	***
6	cadmium	Fcc01428	16S ribosomal RNA gene, no sign hits	VIP	***	***	ns
7	cadmium	Fcc01574	glycine receptor, alpha 1	VIP	***	***	ns
8	cadmium	Fcc01821-1	Isopenicillin N synthetase	VIP	***	***	ns
9	cadmium	Fcc01821-2	Isopenicillin N synthetase	VIP	***	***	ns
10	cadmium	Fcc01910	whey acidic protein	VIP	*	**	ns
11	cadmium	Fcc02275	No Significant Hit	VIP	***	**	***
12	cadmium	Fcc03202	No Significant Hit		ns	ns	ns
13	cadmium	Fcc04239	unknown function		ns	*	ns
14	cadmium	Fcc04580	metalloproteinase; nephrosin; astacin 4		ns	ns	ns
15	cadmium	Fcc04660	No Significant Hit	VIP	ns	ns	ns
16	cadmium	Fcc00074	Cephalosporin hydroxylase		***	ns	ns
17	cadmium	Fcc00343	haloacid dehalogenase-like hydrolase	VIP	*	***	ns
18	cadmium	Fcc00966	Short-chain dehydrogenase/reductase 9		ns	ns	ns
19	cadmium	Fcc00343-2	Haloacid dehalogenase like hydrolase	VIP	*	***	ns
20	cadmium	Fcc00002	Catalase		*	ns	ns
21	cadmium	Fcc00343-1	Haloacid dehalogenase like hydrolase	VIP	ns	***	ns
22	cadmium	Fcc00086	LamininA	VIP	**	**	ns
23	cadmium	Fcc03748	unknown function		**	ns	ns
24	cadmium	Fcc00170	α-aminoadipyl-cysteinyl-valine synthetase		***	***	ns
25	phenanthrene	Fcc05839	alcohol dehydrogenase (acceptor)		ns	ns	ns
26	phenanthrene	Fcc05181	immunoglobulin-like, sid-1-like protein 2		ns	ns	ns
27	phenanthrene	Fcc04834	crossveinless-2; Bmper protein		ns	ns	ns
28	phenanthrene	Fcc03839	LMBR1 domain containing 2		ns	ns	ns
29	phenanthrene	Fcc01609	heat shock protein Hsp70g; HSP68		*	***	ns
30	phenanthrene	Fcc06002	ABC transporter; MOAT-D	VIP	ns	***	***
31	phenanthrene	Fcc03172	putative deoxynucleoside kinase	VIP	ns	ns	ns
32	phenanthrene	Fcc03067	Shprh protein		ns	ns	ns
33	phenanthrene	Fcc04344	laminin, beta 2; laminin S		ns	***	**
34	phenanthrene	Fcc02471	omega class glutathione S-transferase	VIP	ns	ns	ns
35	phenanthrene	Fcc05124	CCCTC-binding factor-like protein	VIP	ns	ns	ns
36	phenanthrene	Fcc04316	phosphoserine aminotransferase		ns	ns	ns
37	phenanthrene	Fcc06085	ABC-type multidrug transport system	VIP	ns	***	***
38	phenanthrene	Fcc03183	scribbled		ns	ns	ns
39	phenanthrene	Fcc01177	ABC transporter ABCC1	VIP	**	***	**
40	phenanthrene	Fcc01609	HSP70; HSP68		**	***	ns
41	phenanthrene	Fcc00015	CYP9/6	VIP	ns	***	***
42	phenanthrene	Fcc00021	Moxd1 protein	VIP	**	***	**
43	phenanthrene	Fcc00058	CYP6N3v2	VIP	ns	ns	ns
44	phenanthrene	Fcc00390	CYP2P3 (fish)		ns	ns	ns

¹ Collembase IDs can be found on http://www.collembase.org

² Important variable in partial least squares differential analysis (VIP); significance levels in two-way ANOVA followed by Bonferroni post hoc testing: ns P > 0.05; * P < 0.05; ** P < 0.01; *** P < 0.001

³ significance levels in two-way ANOVA followed by Bonferroni post hoc testing: ns P > 0.05; * P < 0.05; ** P < 0.01; *** P < 0.001

**Figure 3:**
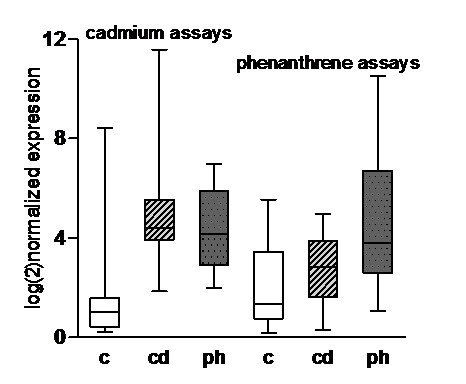
**Accumulated expression levels of cadmium and phenanthrene markers in cadmium and phenanthrene treated *Folsomia candida***. Accumulated average expression level over all markers was upregulated relative to the control for cadmium as well as for phenanthrene (Kruskal-Wallis, χ^2 ^(2) = 40.97, p < 0.0001, followed by Dunn's multiple comparison test). Phenanthrene markers showed higher accumulated expression levels in phenanthrene than cadmium treated samples (paired t test, t (19) = 4.066, p < 0.0001), for cadmium markers no difference between cadmium and phenanthrene samples was detected (paired t test, t (23) = 1.168, p = 0.2). White box = controls; dashed box = cadmium treatment, grey box = phenanthrene treatment.

The accumulated average expression level over all markers was upregulated relative to the control for cadmium as well as for phenanthrene (non parametric testing because of possible collinearity of data; Kruskal-Wallis, χ^2 ^(2) = 40.97, p < 0.0001, followed by Dunn's multiple comparison test), implying that the enrichment for upregulated genes was successful (Figure 3). Phenanthrene markers showed higher accumulated expression levels in phenanthrene than cadmium treated samples (paired t test, t (19) = 4.066, p < 0.0001, Figure 3). For cadmium markers however, no difference between cadmium and phenanthrene treated samples were detected in the accumulated expression levels (paired t test, t (23) = 1.168, p = 0.2, Figure 3), which suggests that the cadmium markers are overall less uniformly expressed than the phenanthrene markers.

**Figure 4:**
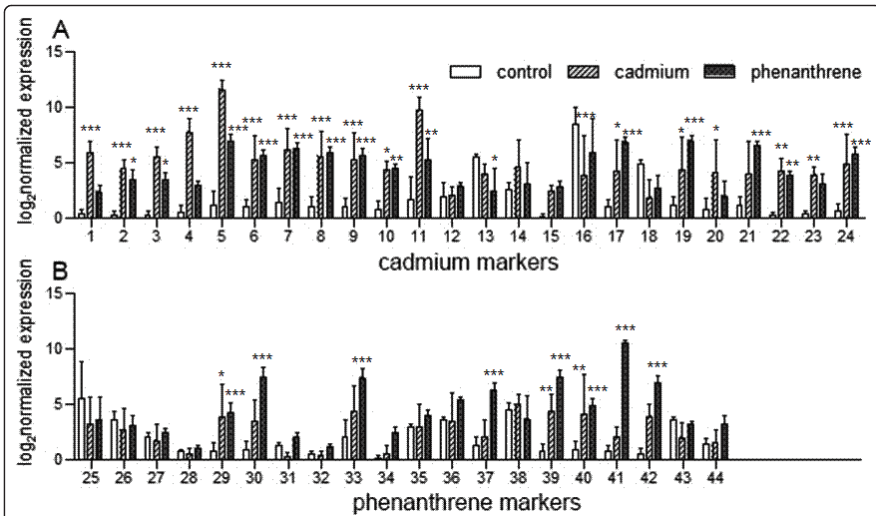
**Relative expression levels of cadmium (a) and phenanthrene (b) markers in cadmium and phenanthrene treated *Folsomia candida***. Significance levels between control and cadmium/phenanthrene: * p < 0.05; ** p < 0.01; *** p < 0.001 (one-way ANOVA; Bonferroni post-hoc test). Details of gene numbers are presented in Table 2.

When looking at the markers individually (two-way ANOVA, F_treatment _(2,43) = 225.1, followed by Bonferroni post hoc testing p < 0.05, Figure 4, Table 2), the majority (18 out of 24) of cadmium markers were significantly regulated by cadmium, but 14 of those were likewise induced by phenanthrene. Surprisingly, two cadmium markers (#13 and 21) were regulated significantly only by phenanthrene. For the phenanthrene markers a smaller proportion (8 out of 20) were significantly upregulated by phenanthrene; four of those also by cadmium.

**Figure 5:**
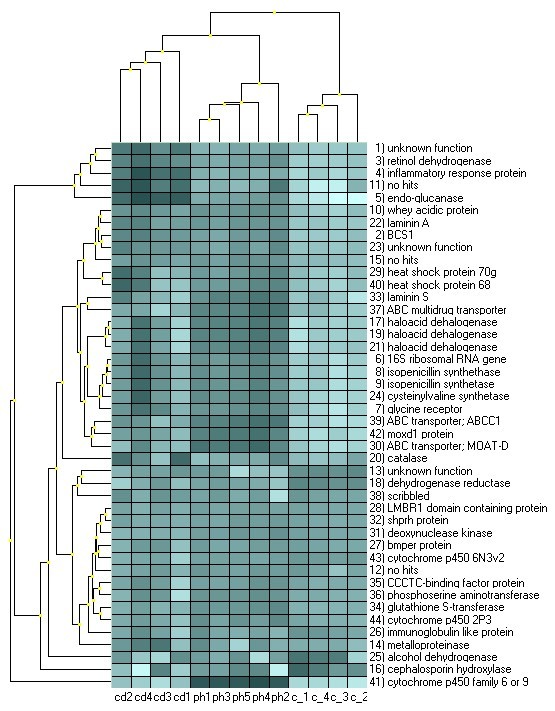
**Hierarchical clustering of mean centered log_2 _normalized expression levels of cadmium and phenanthrene treated *Folsomia candida***. Heatmap view of mean centered expression levels by hierarchical clustering. Darker shading = higher expressions, lighter shading = lower expressions. Dendrograms show the relationships between the samples (columns) and genes (rows) by hierarchical clustering (average linkage of Euclidean distances). Abbreviations of the samples: cd = cadmium treatment, ph = phenanthrene treatment, c = control (water and acetone).

Figure 5 gives an overview of the mean centered expression levels in heatmap format, clustered by hierarchical clustering (average linkage of Euclidean distances) with the darker shading representing higher expressions compared to the lighter shading for lower expressions. The samples (columns) are clustered according to the treatments. A gene cluster of predominantly cadmium-induced assays stands out at the top (rows) including the inflammatory response protein (#4), endo-glucanase and retinol dehydrogenase (#3) as well as two markers with unknown function (#11, 1). The assay most specific to phenanthrene was cytochrome P450 family 6 or 9 (#41), but mainly induced by phenanthrene were also another monooxygenase domain containing protein (#42), three ABC transporters (#30, 37, 39) and Laminin S (#33). A large cluster of genes was indicative for chemical stress in a less compound-specific manner. This included genes involved in antibiotic biosynthesis (#8, 9, 24) the glycine receptor (#7) and heat shock proteins 70 (#29, 40).

### Multimarker Classification

**Figure 6:**
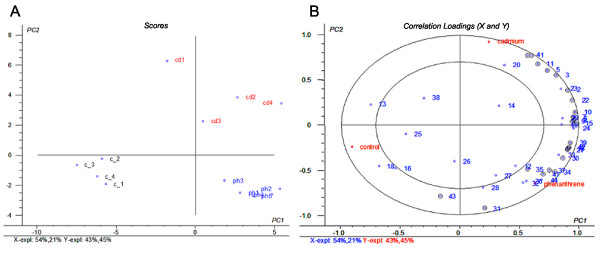
**Scores (A) and Correlation loadings (B) of Partial least squares regression using all markers and training samples**. Scores (A) and Correlation loadings (B) of the first two components of partial least squares regression (PLSR) of 44 gene markers and the training set of cadmium and phenanthrene treated samples of *Folsomia candida*. For details about exposure conditions of the samples and qPCR markers, represented by the numbering in 6B (order as in Table 2), see main text. Abbreviations of the samples in 6A: cd = cadmium treatment, ph = phenanthrene treatment, c = control (water and acetone). 6B shows the correlations between the individual x-loadings (gene expressions) and y-loadings (treatments) for the first two components. Circles indicate the completeness of fit of 100% (outer) and 50% (inner) explained variance [35], calculated by cross validation and Jack-knifing. PLSR analysis was done with The Unscrambler multivariate analysis software [67].

Compound-specific patterns are best recognized when relevant markers are assessed simultaneously. Using multivariate statistics, responses of the markers can be explored and correlated to each other as well as to the treatment. Also it controls the rate of type I error (false positives). Gene expression data was therefore correlated to the treatments by the multivariate method of partial least squares discriminant analysis (PLS-DA) [34], using the samples described above as a training set. The PLS2 regression of the training set showed an optimum model with three principle components (PCs), with an explained variance in the gene expressions of 84.5%. Adding a fourth PC to the model would result in little extra explained variance (4.3%), which is why the simpler model with three PCs was preferred. The scatter plot of scores (Figure 6a) and correlation loadings (Figure 6b) illustrates the decomposition of the first two PCs. Circles in the correlation loadings plot indicate the locus for the 100% (outer) and 50% (inner) explained variance of the individual input variables (completeness of fit) [35]. Controls are separated from the treated samples by PC1, cadmium and phenanthrene exposures are separated by PC2 and 3. Estimation of the uncertainty level on future predictions of unknown samples was done by cross validation and Jack-knifing [35], using the software's standard settings of Martens' Uncertainty Test.

Uncertainties (after validation) reflected by the root mean square error (RMSEP) were 16% for cadmium, 23% for phenanthrene, and 23% for the control group. R^2 ^values (after validation) of the correlations between predicted and known (measured) exposure conditions were respectively 0.90 for cadmium, 0.81 for phenanthrene and 0.79 for the control group.

For each treatment a list of 'important variables' (VIPs) (significant in the uncertainty testing) is calculated from the loading weights and regression coefficients of the x-variables. In the correlation loadings plot (Figure 6b) all 25 VIPs are marked with a black circle. Some genes that were significant in the two-way ANOVA were not selected as VIPs, e.g. the heat shock proteins and laminin S. These markers might be biologically important but may not necessarily be relevant in the PLS-regression (PLSR), presumably because of the presence of other VIPs with a similar expression profile but a lower noise level. Markers that were significant for both treatments, as well as between treatments were all VIPs. There were also several VIPs, such as glutathione S-transferase, that appeared not significant when analyzed independently. Table 2 gives a summary of which markers are labeled as VIPs, as well as their significances resulting from the Bonferroni post hoc tests in the two-way ANOVA.

A test set of samples from a second experiment (De Boer, unpubl. data) was used to evaluate the performance of a PLSR in predicting exposure conditions of independent samples. The model was slightly modified and only the VIPs were included in the calculation, whereas the rest of the markers were included passively, by giving them a very low weight to remove the influence on the model while still showing the correlations to other variables. This did improve the (2PC) model's fit slightly (RMSEP, R^2^: 9%, 0.97; cadmium, 22%, 0.82; phenanthrene, 20%, 0.84; control). Along with a control treatment, these samples included cadmium and phenanthrene treatments which concentrations equaled to the EC_50 _values for reproduction. For phenanthrene, this concentration was lower than those of the samples used for building the model. For cadmium, the concentrations were similar, but two different exposure times (2-days versus 4-days) were tested. Prediction intervals of the predicted value ± RMSEP were used to classify samples. For cadmium, the model was quantitatively able to predict two of the cadmium samples and one of the control samples. No samples were attributed to the phenanthrene class. An alternative approach is to use a 'hard' classification, which assigns each sample to the best fitting class based on the ranking of within class values. This approach resulted in eight out of ten correctly predicted controls, four out of six phenanthrene samples, as well as eight out of ten cadmium samples; the exposure time of the cadmium samples did not affect the ranking of a sample.

## Discussion and Conclusion

### Differences in SSH libraries between cadmium and phenanthrene

Gene expression changes following exposure to chemically spiked media often point to the molecular mechanisms that are used to cope with hazardous substances [6]. Our main purpose in building the SSH libraries was to pick up genes that are regulated as a result of exposure to the chemical compounds. Even though we used different levels of exposure for both chemicals because of changing insights during the course of the study, we were still able to identify toxicant-specific genes. As a result, cross comparison is limited and remains merely qualitative and mechanistically orientated. Here, the molecular responses to cadmium and phenanthrene were investigated in the soil-living indicator organism *F. candida *and stress-related gene fragments were isolated and characterized using Blastx homology queries.

The modest overlap between the SSH and the normalized library [20] affirms that the enrichment in favor of stress-inducible transcripts is a useful step in gene and biomarker discovery, in particular when a transcriptome instead of a complete genome is sequenced.

Our results show that the two chemicals resulted in distinct responses, largely in agreement with existing literature on properties and modes of toxic action of these chemicals. Cadmium is a metal with toxic properties as it interacts with many biochemical targets; its soluble ions have a strong tendency to bind with sulfhydryl (-SH) groups in proteins [11]. One of the main mechanisms of cadmium toxicity is oxidative stress, even though it does not engage in the Fenton reaction [36]. Phenanthrene is an aromatic lipid-soluble compound, and its toxic properties result from its ability to occupy and traverse cell membranes. Metabolism of PAHs through the biotransformation process can generate additional toxic effects through production of reactive oxygen species (ROS), which can cause damage or hamper proper cell functioning due to the high affinity with biomolecules [14]. In mammals, phenanthrene is not metabolized to mutagenic or carcinogenic intermediates, however, its metabolic pathway in invertebrates is not known. We have to take into account that invertebrates may metabolize PAHs to intermediates not seen in vertebrates [37]. Differences in GO Slim annotations of gene fragments found in our SSH libraries in the category of affected cellular compartments related directly to differences in solubility between both chemicals; cadmium effects occurred in the soluble fraction of the cell (intracellular) and phenanthrene effects in the membrane fraction (plasma membrane and smooth endoplasmic reticulum) (Figure 2). The biotransformation process, manifested by the terms metabolism and biosynthesis was the main biological process in the case of phenanthrene. Cadmium showed a more versatile picture, which is not easily explained in generic terms. A summary of the GO EAST analysis is given in DAG format as additional material (Additional files 1, 2, 3).

### Cadmium

#### Uptake and transport

We found several ABC transporters and copper pumps (copper-transporting ATPase 1, 2) induced by cadmium. In yeast, cadmium uptake is provoked by calcium (Ca^2+^) transporters and also vesicular transmembrane processes are mediated by exocytic pathway transporters [38]. We also retrieved a *NIPSNAP1 *homolog, which is known to be an inhibitor of the specific Ca^2+ ^transporter *TRPV6 *[39]. Interestingly, a *NIPSNAP *homolog was identified in an SSH enrichment study conducted for the sister species *Orchesella cincta *[40]. The redox scavenger glutathione has an important role in maintaining cellular redox state [41]. In our study, different glutathione S-transferases were induced and these could be involved with sequestration of cadmium. An important group of proteins that are involved in the protection against oxidative stress are metallothioneins. They bind freely dissolved cadmium ions with extremely high affinity. In many arthropods, like the springtail *Orchesella cincta*, a metallothionein is induced by cadmium [42-44]. It is remarkable that no homologs of metallothionein were picked up during the SSH procedure in *F. candida*. Earlier experiments by our group using degenerated primers neither succeeded in the isolation of a metallothionein gene for *F. candida*. However, very recently Nakamori et al. [45] isolated a metallothionein-like protein in *F. candida*, which appears to be very different from the other collembolan metallothioneins isolated so far.

#### Organelles

In our SSH gene libraries, several mitochondrion related ESTs were enriched (i.e subunits of cytochrome oxidase, NADPH-specific isocitrate dehydrogenase ATP-synthases, Rieske iron-sulfur proteins, *BCS1*) which were linked to GO terms for cellular events of mitochondrial distribution and inheritance. Cannino et al. [46] summarized the direct effects of cadmium on the mitochondrion and oxidative phosphorylation, where cadmium blocks electron flow resulting in uncoupling of the transmembrane proton and voltage gradient which form the proton motive force. Cadmium related uptake kinetics (ABC transporters) force ATP production by oxidative phosphorylation, which result in progressive mitochondrial disruption.

Another enriched term relates to the 26S proteasome complex. We found at least four different subunits induced. Induction of the proteasome complex was found in gene expression studies before, e.g. Nota et al. [17]. Expression of the ubiquitine-mediated pathway for degradation of proteins via the proteasome complex was placed in the context of oxidative stress by Davies [47], however It could also be associated with changes in protein turnover [48].

#### Cell signaling and apoptosis

Due to its valence and chemical characteristics cadmium can imitate calcium and zinc. Induced subunits of Annexin and the acetylcholine receptor are examples where cadmium may have activated expression by mimicking calcium. It may be suggested that some of the cell signaling triggered by cadmium is a primary effect of this mimicking behavior. Supporting evidence of this was found by Roelofs et al. [49]. In a microarray study focused on cadmium tolerance in the springtail *O. cincta *these authors identified five genes participating in phosphatidylinositol and calcium signaling, to be regulated by cadmium treatment, and moreover, that this pathway is involved in cadmium tolerance. Next to a direct interaction, cadmium can induce harm like misfolded proteins or mitochondrial damage, that in turn cause cellular signaling systems to be triggered (e.g. [46,47]). We found the Ras protein signaling to be induced, which is supported by literature (e.g. [50,51]). Like stress-induced signal transduction pathways, also the enriched Vitamin A (retinoid) metabolism can be related to apoptosis [52] by retinoic acid mediated transcriptional activity [53].

### Phenanthrene

#### Uptake and transport

One of the enriched GO terms for phenanthrene was 'plasma membrane'. The primary toxic effect of non polar lipophilic compounds, including PAHs such as phenanthrene, is baseline toxicity (narcosis). This is believed to be the result of reversible and non-specific disturbance of membrane integrity and function resulting from the partitioning of the chemical into biological membranes [6,54]. Metabolism of the accumulated compounds takes place via a biotransformation process in which the compound is first activated, and then conjugated to an endogenous substance, making it ready for excretion in urine or bile.

#### Biotransformation

The majority of enriched GO terms were related to the biotransformation process. For example many retrieved genes relate to the Endoplasmic Reticulum (ER), which is the organelle where the main steps of this process occur. Different glutathione S-transferases involved in chelation of oxygenized metabolites to glutathione, reduce the free flow of reactive metabolites, which is called phase II biotransformation [14]. The last step in the biotransformation was represented by multiple ABC transporters which contribute in the mediated export of bound metabolites via vesicles [14].

### QPCR stress response modeling

The aim of the study was to find biomarkers capable of discriminating the nature of the chemical treatments, as well as the concentration levels of the exposures. The latter was unrealizable within the conditions of this experiment. Further research will therefore aim for the identification of dose related responses, by studying inductions of the markers in response to lower, environmentally relevant, concentration ranges. In acquiring treatment-specific gene fragments SSH proved to be a valuable method. On average, the set of qPCR markers developed from fragments in the SSH libraries were induced by the treatments. A small cluster of markers was found to be cadmium-specific, including an inflammatory response protein and an endo-glucanase. Phenanthrene-specific was cytochrome P450 from family 6 or 9. Lee et al. [55] summarized the role of both these cytochrome families in xenobiotic metabolism. A concentration-dependent induction of *CYP1A*, the vertebrate homolog of the insect xenobiotic biotransformation cytochrome P450 9, was found by Søfteland et al. [56] after PCDD and TCDF exposure of Atlantic salmon hepatocytes.

Using a high throughput qPCR system, made it feasible to measure a set of 44 qPCR markers and perform a multivariate analysis on the expression levels using PLSR. A similar approach was undertaken successfully by Wang et al. in designing a qPCR based application for the prediction of progression of bladder cancer, where a panel of 57 genes resulted in a clinically feasible test [57]. In this study, selection of assays was performed on the basis of microarray profiling, resulting in 50 overexpressed and 15 underexpressed genes. These genes, combined with a set of reference genes and historic markers, were used in a 96-well format low density array card (Applied Biosystems), which allows for multiplex high-throughput QPCR measurements.

We evaluated the diagnostic power of our set of genetic markers by use of a new test set of samples. With an uncertainty level of nearly 20%, the model lacked the capacity to predict particularly the lower concentration phenanthrene treated samples. With validation parameters comparable to the controls, we assume that this was primarily the effect of the concentration difference, rather than the uncertainty level. Molecular methods for ecotoxicological applications in soil must be able to handle the relatively high intrinsic variation in expression data caused by the heterogenic nature of soils and therefore in exposure conditions in test setups. Enhanced predictive power may be acquired when dose response related markers can be included, and the model is trained with samples that include different concentration levels. Noteworthy is that the goodness of fit of the cadmium samples in the test set was not correlated with a difference in exposure time (2 or 4 days). Unfortunately, the effect of exposure time was not tested for phenanthrene.

The possibilities and added value of a multi-marker approach are nicely demonstrated in a study of Ståhlberg et al. [58], who measured alterations in gene expression levels of 15 genes in time after glucose addition, in four different strains of yeast. With the approach they took, the authors classified the genes according to their responses to the treatment and were hence able to validate and interpret responses of some of the less studied genes by several multivariate methods. High throughput qPCR opens up a way for powerful molecular response profiling and functional analysis of the involved genes and interactions between them, which can be an asset for molecular integration in ecotoxicology.

## Methods

### Collembola cultures and exposures

*F. candida *was kept in plastic containers with a water-saturated plaster of Paris bottom containing 10% charcoal at 20°C in a 12:12 light dark regime. The animals were fed dried baker's yeast (Dr. Oetker) ad libitum. For all exposures, adult animals of approximately one month of age were used.

For the SSH library construction, 40-50 exposed adult animals (15 × 3 biological replicates) were used. The level severity of the SSH treatments was to cause reversible and irreversible cell injury and therefore to trigger a diversity of stress controlling pathways. For phenanthrene a high level of effect was chosen, 50% survival over the exposure duration of 7 days (LC_50_;7d), corresponding to a concentration of 840 mmol/kg d.w. LUFA 2.2 reference soil). Surviving animals exposed to this concentration for 7 days were used for the molecular analysis. For cadmium the level of effect was intended not to intervene with the normal biological functioning of the organism on the longer term [59]. Therefore animals were exposed for a period of 48h to 267 μmol/L cadmium (applied in the form of a cadmium chloride solution on cellulose filters, to 50% of the water holding capacity). This concentration corresponds to the pore water concentration of cadmium in comparable soils that results in 50% reduction of reproduction after 28 days of exposure (EC_50 pore water_;28d, for reproduction [60]). Controls, used as 'driver' in the SSH procedure, consisted of an acetone solvent control (phenanthrene) and normal unspiked water control (cadmium). It, however, has to be mentioned that it is extremely difficult to make presumptions on the effects of such 'incident doses' on organism level responses, and therefore we cannot be sure that the actual doses of phenanthrene and cadmium had the considered cellular stress levels.

### RNA isolation, SSH and library construction

Total RNA (isolated with the SV total RNA isolation kit from Promega) was extracted according manufacturer's protocol and pooled for reverse transcription in the SMART cDNA synthesis procedure (Clontech SMART PCR cDNA Synthesis Kit). SSH EST libraries [15,40] were constructed using a PCR-Select cDNA Subtraction Kit (Clontech Laboratories Inc., USA), following the manufactors' protocol. Using a PCR-Select Differential Screening Kit (Clontech Laboratories Inc., USA), a differential screening was employed, following the criteria of the protocol: clones were considered to be differentially expressed when present in a) forward subtracted and unsubtracted tester pool, b) only in the forward subtracted pool (low abundance transcripts enriched during subtraction) c) forward and reverse subtracted pools, when intensity in forward subtraction was > 5 fold compared to reverse subtraction. d) forward and reverse subtracted pools with intensity 3 to 5 fold higher in forward subtracted pool in case the differential response was confirmed by the hybridizations of the unsubtracted pools.

Two times x960 clones, confirmed to be differential were sequenced and processed using the PartiGene pipeline. The clusters were annotated against the BLASTX database of Genbank (expect-value < 10^-5^), followed by GO term assignment when possible (for all further details see [20]).

### Quantitative PCR assay design and validation

From the SSH libraries of phenanthrene and cadmium responsive gene clusters, RT-qPCR assays were designed for clusters of sufficient length (see Additional file 4 for primer constraints and sequence details as well as the associated GO terms). Clusters without significant blastx hit were only used in exceptional cases where the differentiality was extremely high. General assay performance and reaction efficiencies were determined according to previously described procedures [61]. Assays for 19 cadmium and 36 phenanthrene target genes were technically validated using the total RNA from the SSH exposure (see below for qPCR conditions). As internal controls two previously assessed reference genes [61] were used.

### Experimental design

To validate the obtained markers for their capacity in multivariate classification, 4 × 15 adult *F. candida *were exposed on top of a compressed layer soil, to 0.84 mmol/kg d.w. (≈ LC_50_;7d, De Boer unpubl. data) and 0.42 mmol/kg d.w. (≈ LC_50_;28d, [62]) phenanthrene (96h) and 6.9 mmol/kg d.s. (≈LC_50_;28d, [63]) and 1.0 mmol/kg (≈ EC_50_;28d for reproduction, which were reported by Van Gestel and Mol [64] between 0.34 and 0.78 mmol/kg for LUFA 2.2 soil) cadmium (48h). A multivariate PLSR model was calibrated on the basis of these samples as a training set. Validation of the model was done with a test set of additional samples from a separate exposure. Conditions in these exposures were identical to the training experiment described above. Six times fifteen animals were used. The cadmium concentrations were lower than those used in the training set; 0.32 mmol/kg d.s, which corresponds to the measured EC_50_;28d values for reproduction (De Boer unpubl. data) for this experiment. Exposure times were 48h and 96h. For phenanthrene the concentration was lower than the training experiment; 0.22 mmol/kg d.s. (≈ EC_50_;28d for reproduction [62]), with an equal exposure time of 96h.

### cDNA synthesis and high throughput quantitative RT-PCR

Total RNA (SV total RNA isolation kit, Promega) of ten exposed adult animals was pooled and used for reverse transcription with approximately 0.3 μg of total RNA input, using the M-MLV reverse transcriptase (Promega) and oligo d-T primer. cDNA was diluted 5×.

An subset of 44 (24 cadmium, 20 phenanthrene) assays and two reference genes (*YWHAZ*, *SDHA*) (Table 2) was measured by the Biomark high throughput qPCR machine, using 48.48 Dynamic Array chips (Fluidigm). The system as we used it, could measure up to 2304 simultaneous reactions, easily pipetted into the chip setup which looks and handling are comparable to a conventional microtiter plate. Assay and preamplified cDNA mixes are pipetted separately into different inlets. The loading and mixing of each individual sample-assay combination is done by an automated process of high pressure application, which pushes the fluids through a network of micro scale fluid lines into the individual chip wells [10]. Fluorescence measurement by the Biomark works similar to a conventional real-time PCR instrument.

Two slots were left empty as no template control. Preamplification was done according to the manufacturer's protocol (Fluidigm) and diluted 5 times. 1.5 μL preamplified cDNA was mixed together with 4.5 μL sample mix. Sample mix was prepared using 0.6 μL PCR-buffer 20 mM, (Roche); 0.12 μL dNTPs, 10 mM; 0.12 μL FastStart Taq polymerase (Roche); 0.3 μL DA sample loading reagents (Fluidigm); 0.12 μL 50× Rox (Invitrogen); 0.6 μL 10× EvaGreen (Biotum); 2.64 μL ddH_2_O. For the assay mix, 1.8 μL of forward and reverse primers (17 pm/μL) were mixed with 2.5 μL DA assay loading reagents (Fluidigm). QPCR reactions were performed in quadruplets for each sample-assay combination, using a 3-step PCR program (10 min at 95°C; 15 s at 95°C, 30 sec 60°C, 30 sec 72°C, 40 cycles, data acquisition each slice). Assays were validated for this platform before use, by correlating expression levels of samples run on both an Opticon qPCR system (DNA engine 1, MJ Research) and the Biomark qPCR-chip-platform and visual inspection of melting curve analyses and fluorescence curves.

### Computational and statistical analyses

Annotation of the SSH libraries was performed using GO terms generated by Partigene, Collembase [20]. Following, the online GO term classification counter CateGOrizer [27] was used to perform a (single count) counting of the Generic GO Slim ancestor terms [65] for each library, without counting the three root classes (CC, BP and MF). Secondly, an enrichment analysis was performed for each library, as well as a simultaneous comparison between both libraries using GOEAST and multi-GOEAST [28]. A hypergeometric test was used without multiple-test adjustment, therefore the significance level was set more stringently to p = 0.01 for enrichment.

For all quantitative analyses, Genex Light v4.3.5 [66] was used to preprocess the raw qPCR data. The following statistical steps were performed: 1) Averaging of technical qPCR replications. If the standard deviation exceeded 0.5, the fluorescence curves were inspected and in case one of the replicates showed a deviation from the two others it was removed. In all cases the Ct-values consisted of at least two averaged measurements. 2) Efficiency-correction for each assay, 3) Normalization of input using the geometric mean of two reference genes expressions, and 4) log_2 _transformation of the data. Assays that did not perform well in melting curve analysis or showed failure of performance for multiple samples, as well as data with high Ct-values (> 30) were excluded from analysis. Significance of expression levels was determined by ANOVA, using Bonferroni post hoc testing.

Multivariate analyses were performed with The Unscrambler statistical package v9.8 (trial version, [67]). Mean centering of the expressions per assay was applied to the log_2 _data. We used the PLS-DA methods with 24 missing data points filled in during analysis, using the standard setting of the software (PCA as and estimation method for the missing values). The PLS2 model was tested by full cross validation, which involves predicting a portion of the dataset using information from the remainder of the samples [29] and at the same time the software's included Martens' Uncertainty Test was used to assess the stability of the regression results, and to produce uncertainty limits (95% estimated confidence interval [68] for the regression coefficients of each variable.

## Authors' contributions

MEB, JE, DR and NMS conceived the study, set up its design and drafted the manuscript. MEB performed the *F. candida *treatments and conducted the molecular analysis. SB helped in performing the SSH and the differential screening. MJTNT and DR amplified SSH libraries for high throughput EST sequencing and MJTNT processed the raw sequencer trace files. JTD facilitated high throughput qPCR procedure. All authors helped shaping and approved the final manuscript.

## Supplementary Material

Additional file 1 **DAG graph Cellular Compartment**; a multiGOEAST analysis of the cadmium and phenanthrene SSH libraries Click here for file

Additional file 2 **DAG graph Biological Process**; a multiGOEAST analysis of the cadmium and phenanthrene SSH libraries Click here for file

Additional file 3 **DAG graph Molecular Function**; a multiGOEAST analysis of the cadmium and phenanthrene SSH libraries Click here for file

Additional file 4 **Details of the qPCR assays used in this study**; primer sequences and efficiencies; associated GO terms Click here for file

## References

[B1] Chapman RW EcoGenomics - a consilience for comparative immunology? Dev Comp Immunol 2001 25 549 551 10.1016/S0145-305X(01)00045-3 11472776

[B2] Van Straalen NM Roelofs D An Introduction to Ecological Genomics 2006 Oxford: Oxford University Press

[B3] Snell TW Brogdon SE Morgan MB Gene expression profiling in ecotoxicology Ecotoxicology 2003 12 475 483 10.1023/B:ECTX.0000003033.09923.a8 14680327

[B4] Brulle F Morgan AJ Cocquerelle C Vandenbulcke F Transcriptomic underpinning of toxicant-mediated physiological function alterations in three terrestrial invertebrate taxa: A review Environ Pollut 2010 158 2793 2808 10.1016/j.envpol.2010.06.019 20619942

[B5] Steinberg CEW Sturzenbaum SR Menzel R Genes and environment - striking the fine balance between sophisticated biomonitoring and true functional environmental genomics Sci Total Environ 2008 400 142 161 10.1016/j.scitotenv.2008.07.023 18817948

[B6] Ankley GT Bennett RS Erickson RJ Hoff DJ Hornung MW Johnson RD Mount DR Nichols JW Russom CL Schmieder PK Adverse outcome pathways: a conceptual framework to support ecotoxicology research and risk assessment Environ Toxicol and Chem 2010 29 730 741 10.1002/etc.34 20821501

[B7] Martin YC Kofron JL Traphagen LM Do structurally similar molecules have similar biological activity? J Med Chem 2002 45 4350 4358 10.1021/jm020155c 12213076

[B8] Patterson DE Cramer RD Ferguson AM Clark RD Weinberger LE Neighborhood behavior: a useful concept for validation of "molecular diversity" descriptors J Med Chem 1996 39 3049 3059 10.1021/jm960290n 8759626

[B9] Vass L Kelemen JZ Feher LZ Lorincz Z Kulin S Cseh S Dorman G Puskas LG Toxicogenomics screening of small molecules using high-density, nanocapillary real-time PCR Int J Mol Med 2009 23 65 74 19082508

[B10] Spurgeon SL Jones RC Ramakrishnan R High throughput gene expression measurement with real time PCR in a microfluidic Dynamic Array PLoS ONE 2008 3 e1662 10.1371/journal.pone.0001662 18301740 PMC2244704

[B11] Manahan SE Toxicological Chemistry and Biochemistry 2003 Florida: CRC Press

[B12] Brennan RJ Schiestl RH Cadmium is an inducer of oxidative stress in yeast Mutat Res Fund Mol Mech Mut 1996 356 171 178 10.1016/0027-5107(96)00051-6 8841482

[B13] Kültz D Molecular and evolutionary basis of the cellular stress response Annu Rev Physiol 2005 67 225 257 15709958 10.1146/annurev.physiol.67.040403.103635

[B14] Hodgson E (Editor) A Textbook of Modern Toxicology 2004

[B15] Diatchenko L Lau YFC Campbell AP Chenchik A Moqadam F Huang B Lukyanov S Lukyanov K Gurskaya N Sverdlov ED Siebert PD Suppression subtractive hybridization: a method for generating differentially regulated or tissue-specific cDNA probes and libraries Proc Natl Acad Sci USA 1996 93 6025 6030 10.1073/pnas.93.12.6025 8650213 PMC39182

[B16] Ozsolak F Milos PM RNA sequencing: advances, challenges and opportunities Nat Rev Genet 2011 12 87 98 10.1038/nrg2934 21191423 PMC3031867

[B17] Nota B Timmermans M Franken O Montagne-Wajer K Marien J De Boer ME De Boer TE Ylstra B Van Straalen NM Roelofs D Gene expression analysis of collembola in cadmium containing soil Environ Sci Technol 2008 42 8152 8157 10.1021/es801472r 19031917

[B18] Gelfand D Kastury K High-throughput nanovolume qPCR GEN 2009 29 42 43

[B19] Morrison T Hurley J Garcia J Yoder K Katz A Roberts D Cho J Kanigan T Ilyin SE Horowitz D Nanoliter high throughput quantitative PCR Nucleic Acids Res 2006 34 10.1093/nar/gkl639 17000636 PMC1635282

[B20] Timmermans MJTN De Boer ME Nota B De Boer TE Mariën J Klein-Lankhorst RM van Straalen NM Roelofs D Collembase: a repository for springtail genomics and soil quality assessment BMC Genomics 2007 8 341 10.1186/1471-2164-8-341 17900339 PMC2234260

[B21] Genbank http://www.ncbi.nlm.nih.gov/genbank

[B22] Collembase http://www.collembase.org

[B23] Littlefield K Monroe M Venn Diagram Plotter, v1.4.3740 Year of release 2010 http://omics.pnl.gov/software/VennDiagramPlotter.php

[B24] Adhikari BN Wall DH Adams BJ Desiccation survival in an Antarctic nematode: molecular analysis using expressed sequenced tags BMC Genomics 2009 10 69 10.1186/1471-2164-10-69 19203352 PMC2667540

[B25] Matejusova I Felix B Sorsa-Leslie T Gilbey J Noble LR Jones CS Cunningham CO Gene expression profiles of some immune relevant genes from skin of susceptible and responding Atlantic salmon (*Salmo salar L*.) infected with *Gyrodactylus safaris *(Monogenea) revealed by suppressive subtractive hybridisation Int J Parasitol 2006 36 1175 1183 10.1016/j.ijpara.2006.04.009 16806223

[B26] Griffitt RJ Chandler GT Greig TW Quattro JM Cathepsin B and glutathione peroxidase show differing transcriptional responses in the grass shrimp, *Palaemonetes pugio *following exposure to three xenobiotics Environ Sci Technol 2006 40 3640 3645 10.1021/es052537o 16786705

[B27] Hu ZL Bao J Reecy JM CateGOrizer: A Web-Based Program to Batch Analyze Gene Ontology Classification Categories Online J Bioinform 2008 9 108 112

[B28] Zheng Q Wang XJ GOEAST: a web-based software toolkit for Gene Ontology enrichment analysis Nucleic Acids Res 2008 36 W358 W363 10.1093/nar/gkn276 18487275 PMC2447756

[B29] Brereton RG Applied Chemometrics for Scientists 2007 Chichester: Wiley

[B30] Carbon S Ireland A Mungall CJ Shu S Marshall B Lewis S AmiGO: online access to ontology and annotation data Bioinformatics 2009 25 288 289 10.1093/bioinformatics/btn615 19033274 PMC2639003

[B31] Bochtler M Ditzel L Groll M Hartmann C Huber R The proteasome Annu Rev Biophys Biomol Struct 1999 28 295 317 10.1146/annurev.biophys.28.1.295 10410804

[B32] Aharonowitz Y Cohen G Martin JF Penicillin and cephalosporin biosynthetic genes - structure, organization, regulation and evolution Annu Rev Microbiol 1992 46 461 495 10.1146/annurev.mi.46.100192.002333 1444264

[B33] Nota B Bosse M Ylstra B van Straalen NM Roelofs D Transcriptomics reveals extensive inducible biotransformation in the soil-dwelling invertebrate *Folsomia candida *exposed to phenanthrene BMC Genomics 2009 10 13 10.1186/1471-2164-10-236 19457238 PMC2688526

[B34] Wold S Sjostrom M Eriksson L PLS-regression: a basic tool of chemometrics Chemom Intell Lab Syst 2001 58 109 130 10.1016/S0169-7439(01)00155-1

[B35] Martens H Martens M Modified Jack-knife estimation of parameter uncertainty in bilinear modelling by partial least squares regression (PLSR) Food Qual Preference 2000 11 5 16 10.1016/S0950-3293(99)00039-7

[B36] Valko M Morris H Cronin MTD Metals, toxicity and oxidative stress Curr Med Chem 2005 12 1161 1208 10.2174/0929867053764635 15892631

[B37] Stroomberg GJ Zappey H Steen R van Gestel CAM Ariese F Velthorst NH Van Straalen NM PAH biotransformation in terrestrial invertebrates - a new phase II metabolite in isopods and springtails Comp Biochem Physiol C: Toxicol Pharmacol 2004 138 129 137 10.1016/j.cca.2004.06.004 15450860

[B38] Lauer CM Bonatto D Mielniczki-Pereira AA Schuch AZ Dias JF Yoneama ML Henriques JAP The PMR1 protein, the major yeast Ca^2+^-ATPase in the Golgi, regulates intracellular levels of the cadmium ion Fems Microbiol Lett 2008 285 79 88 10.1111/j.1574-6968.2008.01214.x 18510555

[B39] Schoeber JPH Topala CN Lee KP Lambers TT Ricard G Van der Kemp A Huynen MA Hoenderop JGJ Bindels RJM Identification of NIPSNAP1 as a novel auxiliary protein inhibiting TRPV6 activity Pflugers Arch 2008 457 91 101 10.1007/s00424-008-0494-5 18392847

[B40] Roelofs D Marien J van Straalen NM Differential gene expression profiles associated with heavy metal tolerance in the soil insect *Orchesella cincta* Insect Biochem Mol Biol 2007 37 287 295 10.1016/j.ibmb.2006.11.013 17368192

[B41] Jamieson DJ Oxidative stress responses of the yeast *Saccharomyces cerevisiae* Yeast 1998 14 1511 1527 10.1002/(SICI)1097-0061(199812)14:16<1511::AID-YEA356>3.0.CO;2-S 9885153

[B42] Timmermans M Ellers J Roelofs D Van Straalen NM Metallothionein mRNA expression and cadmium tolerance in metal-stressed and reference populations of the springtail *Orchesella cincta* Ecotoxicology 2005 14 727 739 10.1007/s10646-005-0020-x 16160751

[B43] Sterenborg I Roelofs D Field-selected cadmium tolerance in the springtail *Orchesella cincta *is correlated with increased metallothionein mRNA expression Insect Biochem Mol Biol 2003 33 741 747 10.1016/S0965-1748(03)00070-5 12826101

[B44] Haq F Mahoney M Koropatnick J Signaling events for metallothionein induction Mutat Res 2003 533 211 226 14643422 10.1016/j.mrfmmm.2003.07.014

[B45] Nakamori T Fujimori A Kinoshita K Ban-nai T Kubota Y Yoshida S mRNA expression of a cadmium-responsive gene is a sensitive biomarker of cadmium exposure in the soil collembolan Folsomia candida Environ Pollut 2010 158S 1689 1695 10.1016/j.envpol.2009.11.022 20022415

[B46] Cannino G Ferruggia E Luparello C Rinaldi AM Cadmium and mitochondria Mitochondrion 2009 9 377 384 10.1016/j.mito.2009.08.009 19706341

[B47] Davies KJA Degradation of oxidized proteins by the 20S proteasome Biochimie 2001 83 301 310 10.1016/S0300-9084(01)01250-0 11295490

[B48] Hochstrasser M Ubiquitin, proteasomes, and the regulation of intracellular protein degradation Curr Opin Cell Biol 1995 7 215 223 10.1016/0955-0674(95)80031-X 7612274

[B49] Roelofs D Janssens TKS Timmermans M Nota B Marien J Bochdanovits Z Ylstra B Van Straalen NM Adaptive differences in gene expression associated with heavy metal tolerance in the soil arthropod *Orchesella cincta* Mol Ecol 2009 18 3227 3239 10.1111/j.1365-294X.2009.04261.x 19566677

[B50] Valbonesi P Ricci L Franzellitti S Biondi C Fabbri E Effects of cadmium on MAPK signalling pathways and HSP70 expression in a human trophoblast cell line Placenta 2008 29 725 733 10.1016/j.placenta.2008.05.004 18571719

[B51] Kamata H Tanaka C Yagisawa H Matsuda S Gotoh Y Nishida E Hirata H Suppression of nerve growth factor-induced neuronal differentiation of PC12 cells - N-acetylcysteine uncouples the signal transduction from Ras to the mitogen-activated protein kinase cascade J Biol Chem 1996 271 33018 33025 10.1074/jbc.271.41.25611 8955147

[B52] Gottlieb RA Programmed cell death Drug News Perspect 2000 13 471 476 12937619

[B53] Cui YX Freedman JH Cadmium induces retinoic acid signaling by regulating retinoic acid metabolic gene expression J Biol Chem 2009 284 24925 24932 10.1074/jbc.M109.026609 19556237 PMC2757195

[B54] Escher BI Hermens JLM Modes of action in ecotoxicology: their role in body burdens, species sensitivity, QSARs, and mixture effects Environ Sci Technol 2002 36 4201 4217 10.1021/es015848h 12387389

[B55] Lee SH Kang JS Min JS Yoon KS Strycharz JP Johnson R Mittapalli VM Margam VM Sun W Li HM Decreased detoxification genes and genome size make the human body louse an efficient model to study xenobiotic metabolism Insect Mol Biol 2010 19 5 599 615 10.1111/j.1365-2583.2010.01024.x 20561088 PMC2944910

[B56] Søfteland L Eide I Olsvik PA Factorial design applied for multiple endpoint toxicity evaluation in Atlantic salmon (*Salmo salar L*.) hepatocytes Toxicol in Vitro 2009 23 1455 1464 19607907 10.1016/j.tiv.2009.07.014

[B57] Wang R Morris DS Tomlins SA Lonigro RJ Tsodikov A Mehra R Giordano TJ Kunju LP Lee CT Weizer AZ Chinnaiyan AM Development of a multiplex quantitative PCR signature to predict progression in non-muscle-invasive bladder cancer Cancer Res 2009 69 3810 3818 10.1158/0008-5472.CAN-08-4405 19383904 PMC2713755

[B58] Ståhlberg A Elbing K Andrade-Garda JM Sjogreen B Forootan A Kubista M Multiway real-time PCR gene expression profiling in yeast *Saccharomyces cerevisiae *reveals altered transcriptional response of ADH-genes to glucose stimuli BMC Genomics 2008 9 15 18412983 10.1186/1471-2164-9-170 PMC2335116

[B59] Andersen ME Dennison JE Thomas RS Conolly RB New directions in incidence-dose modeling Trends Biotechnol 2005 23 122 127 10.1016/j.tibtech.2005.01.007 15734554

[B60] Van Gestel CAM Koolhaas JE Water-extractability, free ion activity, and pH explain cadmium sorption and toxicity to *Folsomia candida *(Collembola) in seven soil-pH combinations Environ Toxicol Chem 2004 23 1822 1833 10.1897/03-393 15352469

[B61] De Boer ME De Boer TE Marien J Timmermans M Nota B van Straalen NM Ellers J Roelofs D Reference genes for QRT-PCR tested under various stress conditions in *Folsomia candida *and *Orchesella cincta *(Insecta, Collembola) BMC Mol Biol 2009 10 54 10.1186/1471-2199-10-54 19486513 PMC2698932

[B62] Droge STJ Paumen ML Bleeker EAJ Kraak MHS van Gestelt CAM Chronic toxicity of polycyclic aromatic compounds to the springtail *Folsomia candida *and the enchytraeid *Enchytraeus crypticus* Environ Toxicol Chem 2006 25 2423 2431 10.1897/05-628R.1 16986798

[B63] Van Gestel CAM Van Diepen AMF The influence of soil moisture content on the bioavailability and toxicity of cadmium for *Folsomia candida *Willem (Collembola: Isotomidae) Ecotoxicol Environ Saf 1997 36 123 132 10.1006/eesa.1996.1493 9126429

[B64] Van Gestel CAM Mol S The influence of soil characteristics on cadmium toxicity for *Folsomia candida *(Collembola: isotomidae) Pedobiologia 2003 47 387 395 10.1078/0031-4056-00202

[B65] GO Slim and Subset Guide http://www.geneontology.org/GO.slims.shtml

[B66] MultiD Analyses AB GenEx Light Software MultiD Analyses AB 2008 http://www.multid.se version 4.3.5, trial version 4.4.2 Pro

[B67] CAMO ASA The Unscrambler Software CAMO ASA 2009 http://www.camo.com version 9.8, trial edition

[B68] CAMO ASA The Unscrambler User Manual http://www.camo.com

[B69] Chart Tool http://www.onlinecharttool.com

