# Supplementary figures and images for: High throughput nano-liter RT-qPCR to classify soil contamination using a soil arthropod
Source: BMC Mol Biol. 2011 Mar 1;12:11. doi: 10.1186/1471-2199-12-11 (PMC3060125; doi:10.1186/1471-2199-12-11)

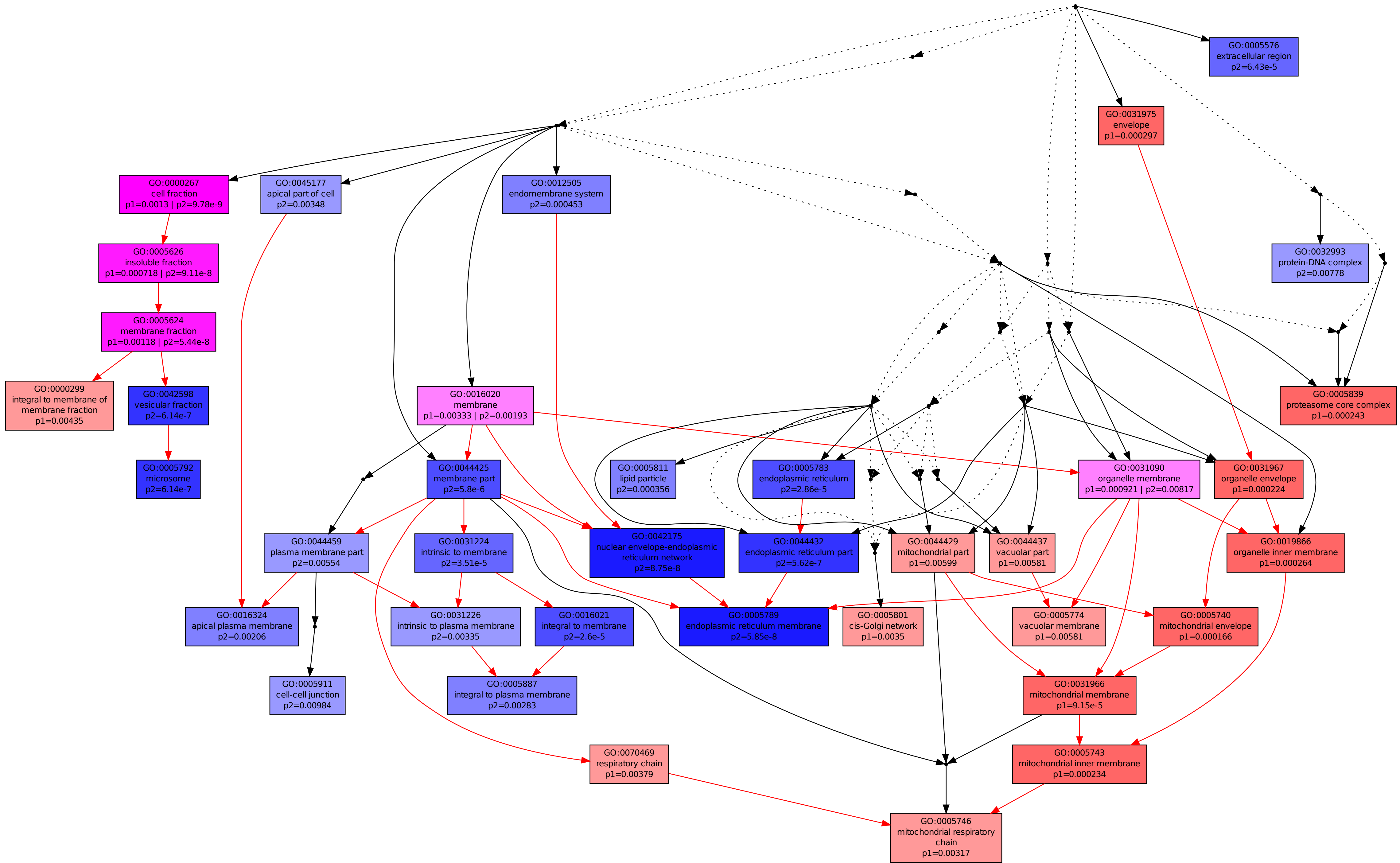

Supplement: Additional file 1 — DAG graph Cellular Compartment; a multiGOEAST analysis of the cadmium and phenanthrene SSH libraries [file 1471-2199-12-11-S1.PDF]

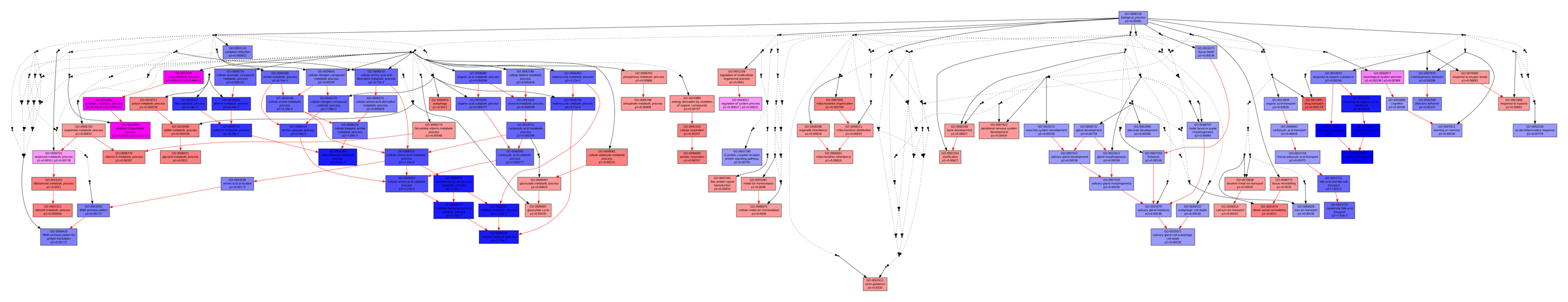

Supplement: Additional file 2 — DAG graph Biological Process; a multiGOEAST analysis of the cadmium and phenanthrene SSH libraries [file 1471-2199-12-11-S2.PDF]

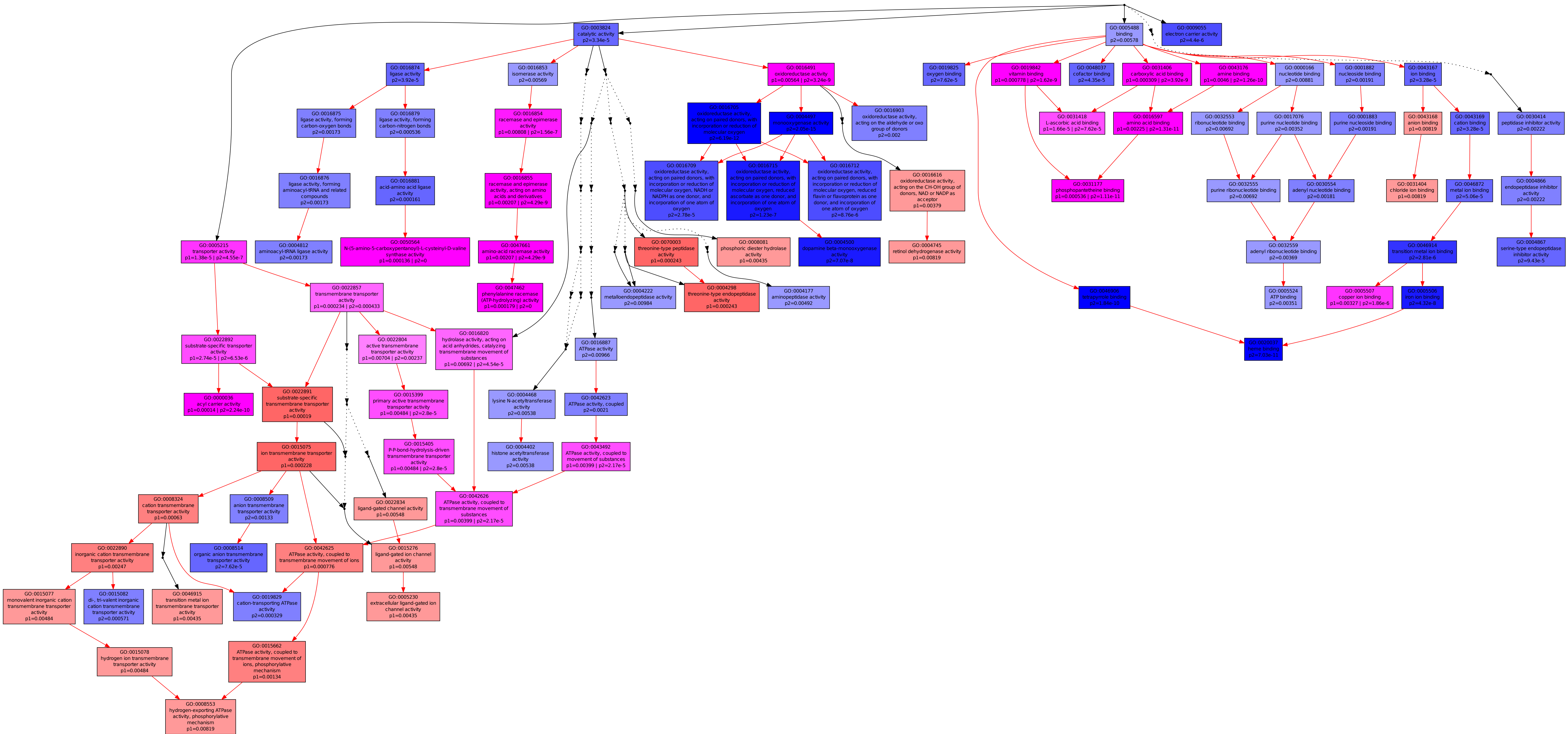

Supplement: Additional file 3 — DAG graph Molecular Function; a multiGOEAST analysis of the cadmium and phenanthrene SSH libraries [file 1471-2199-12-11-S3.PDF]
